# A mathematical model describes the malignant transformation of low grade gliomas: Prognostic implications

**DOI:** 10.1371/journal.pone.0179999

**Published:** 2017-08-01

**Authors:** Magdalena U. Bogdańska, Marek Bodnar, Monika J. Piotrowska, Michael Murek, Philippe Schucht, Jürgen Beck, Alicia Martínez-González, Víctor M. Pérez-García

**Affiliations:** 1 Faculty of Mathematics, Informatics and Mechanics, University of Warsaw, Warsaw, Poland; 2 Departamento de Matemáticas, Universidad de Castilla-La Mancha, Ciudad Real, Spain; 3 Universitätsklinik für Neurochirurgie, Bern University Hospital, Bern, Switzerland; Universidad de Navarra, SPAIN

## Abstract

Gliomas are the most frequent type of primary brain tumours. Low grade gliomas (LGGs, WHO grade II gliomas) may grow very slowly for the long periods of time, however they inevitably cause death due to the phenomenon known as the malignant transformation. This refers to the transition of LGGs to more aggressive forms of high grade gliomas (HGGs, WHO grade III and IV gliomas). In this paper we propose a mathematical model describing the spatio-temporal transition of LGGs into HGGs. Our modelling approach is based on two cellular populations with transitions between them being driven by the tumour microenvironment transformation occurring when the tumour cell density grows beyond a critical level. We show that the proposed model describes real patient data well. We discuss the relationship between patient prognosis and model parameters. We approximate tumour radius and velocity before malignant transformation as well as estimate the onset of this process.

## Introduction

Gliomas are the most frequent type of brain tumours, as they represent approximately 30% of all central nervous system tumours and about 80% of all malignant brain tumours [1]. The term “gliomas” refers to tumours originating from glial cells (mainly from astrocytes and oligodendrocytes) and includes astrocytomas, oligodendrogliomas and tumours which have features of both astrocytoma and oligodendroglioma (hence called oligoastrocytomas). They can be further separated into different histologic grades according to their morphologic features reflecting their natural history or biologic behaviour. According to the World Health Organisation (WHO) grade I astrocytomas (pilocytic astrocytomas) are very rare, non-infiltrating and usually curable, thus will not be addressed in this article. WHO grade II gliomas are usually referred to as low-grade gliomas (LGGs), while WHO grade III and IV—as high grade gliomas (HGGs) (see [2] for the details of the classification).

LGGs are incurable primary tumours, usually occurring in frontal and temporal lobes. Typically LGG patients present only epileptic seizures, while other symptoms (headaches, lethargy, mental changes) are less common. Usually LGGs are slowly-growing, infiltrative tumours, but the prognoses for LGGs patients are diverse. Some of those tumours grow very slowly for years, while others progress rapidly causing major neurological deficits and subsequent death. Because of the unpredictable clinical course, treatment strategies for LGG vary from the “wait and see” approach to gross total resection followed by immediate radiotherapy or chemotherapy. Reports [3–5] provide a strong support for the early use of surgery as it influences the time to progression and median survival. However, due to the infiltrative nature of gliomas, surgery alone is able to eradicate only the tumour bulk and thus other therapeutic treatments are necessary to try to control the disease. Radiotherapy has been usually deferred in LGG patients due to its toxicity and moderate impact on patients overall survival [6–8]. Currently it is most frequently used only for patients with fast growing tumours or with significant enhancement on post-contrast T1 magnetic resonance imaging (MRI), see for instance [9]. As to chemotherapy, the preferred chemotherapeutic agent, temozolomide, is being used because of its effectiveness and limited side effects [10–12]. However the treatment of LGGs is controversial and in general the decision on the individual treatment strategy is based on numerous factors such as patient’s age at diagnosis, performance status, patient preference and tumour location [9].

Most LGG patients die due to the transformation of the tumour into a higher grade one, which is a process known as malignant transformation, anaplastic transformation or malignant progression. Median survival of LGG patients is between 5 and 10 years [13], compared with one to two years for HGG patients [14].

The time of occurrence of malignant transformation differs among patients. The results vary among clinical studies with a 5-year malignancy-free survival rates (that is time when malignant transformation does not occur) from 30 to 70% [15–19]. There are also reports claiming that all LGGs undergo malignant transformation during their clinical course, *e.g.* [20, 21]. Radiologically, malignant transformation is usually defined based on the notable appearance of contrast enhancement on MRI and/or a histopathologically proven malignant degeneration in tissue acquired during biopsy or resection [18].

It was reported that LGGs displaying preoperative contrast enhancement had a significant increased risk of recurrence [18], thus the complete resection of contrast enhancement areas of the tumour significantly increases time to phenotypic change [22]. Medical doctors believe that early detection of indicators of malignant transformation could improve the prognosis, suggesting the radiological verification of relative cerebral blood volume [22, 23], the pathology of gemistocytic astrocytoma [24], the overexpression of epidermal growth factor (EDGF) and the absence of p53 mutation [25]. However, the statistical significance of such indicators is still under study.

There has been a lot of activity on mathematical modelling of LGGs in the last years, *e.g.* [26–33]. LGGs do not present metastasis, aberrant angiogenesis, hypoxia or necrosis. Thus, we will not incorporate these elements in the model and instead we build a simple continuous mathematical model with a minimal number of parameters. To validate our model we use quantitative measurements of LGGs growth rates as suggested in [34].

## Materials and methods

### Mathematical model

Our mathematical model describes the change of the tumour cell density in time and space due to the interplay of net proliferation and net diffusion of cancer cells, as in some previous works [35, 36].

It has been suggested that malignant transformation of LGGs may be induced by a high cell density focus [26, 29, 37–39]. As a result, tumour cells may start having a limited access to nutrients causing major changes in the tumour microenvironment, including vessels damage, generation of hypoxic foci, stabilization of hypoxia-dependent signalling molecules like hypoxia-inducible factor-1 (HIF-1) and increase of genomic instability [38, 40–42]. These changes lead to the appearance of more aggressive tumour cell phenotypes and/or additional mutations.

Thus we base our model of malignant transformation on the assumption that the first step in this phenotypic transition is the growth of the tumour density beyond a certain critical level *L*_*crit*_ initiating a non-reversible damage to the microenvironment [39]. Beyond that point hypoxia arises and angiogenesis is triggered. However, this microvasculature is aberrant and leads to both chronic and acute hypoxia events. This abnormal vasculature plays a key role in the development of more aggressive phenotypes [42, 43] and an enhanced genetic instability.

Malignant transformation cannot be reversed, once the transformation is triggered cells can not change their phenotype to a less aggressive one because of the accumulation of new mutations. We assume that after the onset of malignant transformation cells take some time *τ* to acquire a more aggressive high grade behaviour.

Cells before and after transformation differ in dynamic properties, which is reflected in the model by different proliferation and motility rates (*ρ*_*L*_, *D*_*L*_ and *ρ*_*H*_, *D*_*H*_ for LGG and HGG, respectively).

The density of LGG cells is described by a non-negative function $L:\left[0,+\infty \right)\times \Omega \to R_{+}$, where Ω = [−*B*, *B*] describes the brain domain under consideration. The spatio-temporal density of the more malignant (transformed) cells is described by a function $H:\left[0,+\infty \right)\times \Omega \to R_{+}$. Then, the full mathematical model for the evolution of both tumour cells populations is given by the following system of partial differential equations:

$$\frac{\partial L}{\partial t}=\rho_{L}L(1-\frac{L+H}{K})+D_{L}\Delta L-\frac{1}{\tau }S_{LH}\left(L+H\right)L,$$ (1a)

$$\frac{\partial H}{\partial t}=\rho_{H}H(1-\frac{L+H}{K})+D_{H}\Delta H+\frac{1}{\tau }S_{LH}\left(L+H\right)L,$$ (1b)

with initial conditions:

$$L\left(0,x\right)=L_{0}\left(x\right)\in C^{2}(\bar{\Omega }),\;H\left(0,x\right)=0,$$ (1c)

and no-flux boundary conditions:

$$\frac{\partial L}{\partial n}{|}_{\partial \Omega }=\frac{\partial H}{\partial n}{|}_{\partial \Omega }=0.$$ (1d)

Clearly, system (1) is a pair of Fisher–Kolmogorov–type equations (FKEs) [35]. The last term in both equations describes the malignant transformation of LGG cells into HGG cells inspired by [42]. In the proposed model this is described by continuous switch function $S_{LH}:R_{+}\to \left[0,1\right]$ depending on the total cell density and the rate 1/*τ* and having the following form:

$$S_{LH}\left(T\right)=\{\begin{array}{ll} 0 & \text{for }T<L_{crit}-\Delta_{crit}\\ 0.5\;\left[1+\coth\left(1\right)\tanh\left(\frac{T-L_{crit}}{\Delta_{crit}}\right)\right] & \text{for }T\in \left[L_{crit}-\Delta_{crit},L_{crit}+\Delta_{crit}\right]\\ 1 & \text{for }T>L_{crit}+\Delta_{crit}, \end{array}$$ (2)

where *T* is the total tumour density, *L*_*crit*_ is the density of tumour cells triggering malignant transformation and Δ_*crit*_ is the width (or sensitivity) of the switch function. In what follows, we express *L*_*crit*_ in terms of the maximal cellular density *K*, that is *L*_*crit*_ = *βK* for some *β* ∈ (0, 1).

#### Initial data

As in [35] we assume that initial cells density distribution is a Gaussian one with a mean cell density *h*_0_ at the centre of the tumour *x* = 0, *i.e.*

$$\begin{array}{c} L_{0}\left(x\right)=h_{0}\exp(-\frac{x^{2}}{\sigma }), \end{array}$$ (3)

where *σ* is a measure of the spread of LGG cells.

#### Typical values and ranges of the model parameters

System (1) has eight parameters describing the dynamical properties of the two glioma cells compartments and the phenomenon of malignant transformation. Typical values together with the references used in this paper are summarised in Tables 1 and 2.

**Table 1:** Typical parameter values for system (1).

Param.	Description	Value	Units	References
*K*	carrying capacity (maximal cellular density)	10^8^	cells/mm^3^	[44]
*ρ*_*H*_	proliferation rate of HGG cells	0.042	1/day	[36, 45]
*d*	detection threshold	0.16K	cells/mm^3^	[26, 37, 46]
*L*_*crit*_	tumour cell density causing malignant transformation	0.6*K*	cells/mm^3^	[30]
Δ_*crit*_	variation in density *L*_*crit*_	0.05*L*_*crit*_	cells/mm^3^	Assumed
*τ*	time of change to HGG phenotype	100	day	Estimated

**Table 2:** Ranges of fitted parameters for system (1).

Param.	Description	Range	Units	References
*h*_0_	initial mean LGG cell density	0.3*K*–0.57*K*	cells/mm^3^	[30]
*ρ*_*L*_	proliferation rate of LGG cells	0.0001–0.008	1/day	[28, 36]
*D*_*L*_	diffusion rate of LGG cells	0.0003–0.008	mm^2^/day	[28]
*D*_*H*_	diffusion rate of HGG cells	0.0008–0.9	mm^2^/day	[36, 46–48]

The maximal tumour density *K* is estimated by taking the typical astrocyte size to be about 10 *μ*m in diameter leading to a value 10^8^cells/mm^3^ [44, 46].

The parameters *ρ*_*L*_, *D*_*L*_ and *ρ*_*H*_, *D*_*H*_ quantify the overall biological aggressiveness of gliomas growth, *e.g.* proliferation rates *ρ*_*L*_ and *ρ*_*H*_ are based on the observable values of tumour cells doubling times. We assume LGG proliferation rate *ρ*_*L*_ to be larger than 0.0001/day, which is a value 10 times smaller than the smallest proliferation rate observed in study of Gerin *et al.* [28]. As an upper bound for *ρ*_*L*_ we take a value 0.008/day, which is the smallest value of proliferation rate observed for HGGs [36]. The diffusion coefficient for LGGs is chosen in the range between 0.0003 and 0.008mm^2^/day. These values are, respectively, around three times smaller than minimal and three times larger than the maximal values for LGG diffusion coefficients estimated in [28]. For HGG cells, we assume that they proliferate with a typical rate 0.042/day observed in this kind of tumours, see *e.g.* [36, 45] and move with diffusion coefficient between 0.0008 and 0.9mm^2^/day. These values are close to minimal and maximal diffusion rates estimated in [36].

The value of critical density *L*_*crit*_ triggering microenvironment damage and the malignant transformation is taken to be around 60% of the carrying capacity *K* in agreement with previous estimates [29, 30]. The switch function sensitivity Δ_*crit*_ is arbitrary chosen to be 5%.

The time *τ* needed for a high grade tumour to arise corresponds to the time required for the development of hypoxia in the presence of a high cellularity, the generation of transient hypoxic events leading to the development of more aggressive phenotypes and higher genetic instability leading to new mutations. Since typical angiogenesis times are of the order of 1-2 weeks we can estimate *τ* to be of the order of a few months. Thus, we assume that typical values of *τ* should be in the range 100-200 days.

The tumour cell density *h*_0_ leading to relevant symptoms and disease detection is difficult to estimate as it can vary a lot depending on the tumour location. The normal physiological value of cellularity of brain tissue is around 10-15%. LGGs are characterised, among others, by an increased cellularity with respect to the healthy brain tissue. We will assume that the minimal mean density leading to glioma diagnosis is around 0.3*K* as in [30]. It means that the symptoms occur when the tumour cells density is 30% of the maximal tissue density. Then we can impose the initial mean density *h*_0_ to be no smaller than 0.3*K*. On the other hand, as we consider only tumours before transformation, see Eq (3), this value should be naturally smaller than the value 0.57*K*, which corresponds to minimal density causing the onset of LGG cells transformation as discussed previously.

### Patients data

A retrospective study of the volumetric growth of LGGs was developed to verify the potential of the mathematical model to describe the malignant transformation. Initially, for the presented study 82 patients diagnosed with LGG and followed with MRI scans at the Bern University Hospital between 1990 and 2013 were considered. The study was approved by Kantonale Ethikkommission Bern (Bern, Switzerland), the approval number: 07.09.72. The data was analysed anonymously.

The criteria for inclusion of the patients into study were: (i) first biopsy/surgery confirmed LGG (astrocytoma, fibrillary astrocytoma, oligoastrocytoma or oligodendroglioma), (ii) second biopsy/surgery confirmed HGG (anaplastic oligodendroglioma, anaplastic astrocytoma, anaplastic oligoastrocytoma or glioblastoma), (iii) availability of at least 5 MRI scans before the histological confirmation of the malignant transformation, (iv) no treatment given in the period of study and (v) no decrease of total tumour size observed in the absence of treatment. Among all considered patients, 32 had confirmed malignant transformation and 8 satisfied all of the inclusion criteria of the study. Table 3 summarises the included patients data.

**Table 3:** Characteristics of patients selected in the study.

Age at diagnosis, mean (st. deviation),	37.89 (13.66)
Sex, M/F	3/5
*Histology at diagnosis*	
Oligodendroglioma	2
Oligoastrocytoma	2
Astrocytoma	3
Fibrillary astrocytoma	1
*Ki-67 LI at diagnosis, mean (st. deviation)*	4.71% (1.72%)
*Histology after malignant transformation*	
Anaplastic oligodendroglioma	4
Anaplastic astrocytoma	4
*Ki-67 LI after malignant transformation, mean (st. deviation)*	14.25% (4%)

### Radiological measurements of tumour size

Radiological glioma growth was quantified by the measurements of the tumour diameter on successive T2 (or FLAIR) MRI scans. The three largest tumour diameters (*D*_1_, *D*_2_, *D*_3_) according to three reference orthogonal planes (axial, coronal and sagittal) were measured and the tumour volumes were estimated using the ellipsoidal approximation: *V* = (*D*_1_ ⋅ *D*_2_ ⋅ *D*_3_)/2, following the standard clinical practice [34, 49]. Then the mean tumour diameter (MTD) was calculated from the tumour volume *V* using the equation *MTD* = (2*V*)^1/3^.

In T2/FLAIR sequences the delineated abnormality corresponds to the presence of oedema, see [50]. In LGGs, oedema correlates locally with the presence of glioma cells [37]. We assumed, in line with previous works [26, 46, 51], that the T2/FLAIR signal is detectable above a certain local cell-density threshold. The analysis of biopsies in LGG patients suggests that the detection threshold for gliomas should be fixed between 10 and 20% of the maximal local tissue density *K* [37]. In the following, similarly to [46, 52] we assumed that the threshold of detection of gliomas *d* equals 0.16*K*, what allows to calculate the diameter of the radiologically detectable part of simulated tumour due to system (1).

### Computational details and model fitting

Longitudinal volumetric patient data was used to fit the parameters of system (1). Specifically, *h*_0_, *ρ*_*L*_, *D*_*L*_ and *D*_*H*_ were considered to be patient-specific and thus fitted for each patient. The remaining parameters were chosen as described in Sec. Typical values and ranges of the model parameters.

We fixed the initial condition (3) on the basis of the first MRI scan for each patient. Namely for each patient the variance of LGG cells distribution was computed through

$$\begin{array}{c} \sigma =-r_{0}^{2}/\ln(\frac{d}{h_{0}}), \end{array}$$ (4)

where *r*_0_ is the radius of the tumour calculated from the first MRI scan considered in the study, *d* is the detection threshold and *h*_0_ is the fitted mean cell density.

System (1) was simulated using the standard Matlab PDE solver *pdepe*. Since the bulk dynamics of FK-type equations does not depend much on the spatial dimensionality (see [43] for a similar example) we chose to simulate model equations in one dimensional domain. In order to avoid the boundary effects and focus on the dynamics of the tumour bulk, we have taken the computational domain Ω to be 10 cm which is much larger than the typical tumour size. The error between measured tumour sizes and model outputs for each patient was based on the least squares method. The fitted parameters were obtained using particle swarm optimization algorithm, originally contributed to Kennedy, Eberhart and Shi [53, 54] and implemented in Matlab with a constriction factor introduced by Clerc and Kennedy [55]. For the purpose of fitting LGGs evolution 100 iterations of this algorithm were computed for each patient and the size of swarm in each iteration step was set to be 100. For each patient the set of fitted parameters (*h*_0_, *ρ*_*L*_, *D*_*L*_, *D*_*H*_) were fitted at once with starting point chosen visually.

## Results

### Evolution of virtual patients’ tumours

**Fig 1:**
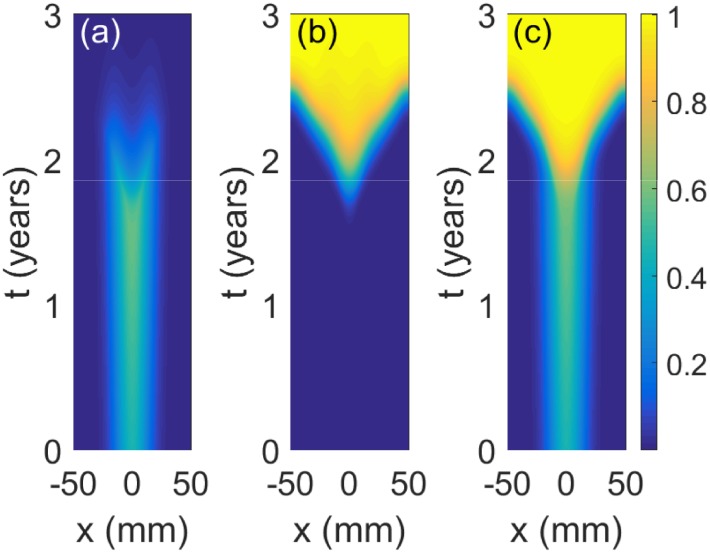
Spatiotemporal simulations of the malignant transformation of LGGs. Pseudocolor plots represent densities of (a) LGG cells, (b) HGG cells and (c) total (LGG + HGG) population with maximal density rescaled to 1. The vertical and horizontal axes correspond to time in years and space in mm, respectively. The virtual tumour evolves according to system (1) with initial condition and the values of parameters as in Sec. Evolution of virtual patients’ tumours.

**Fig 2:**
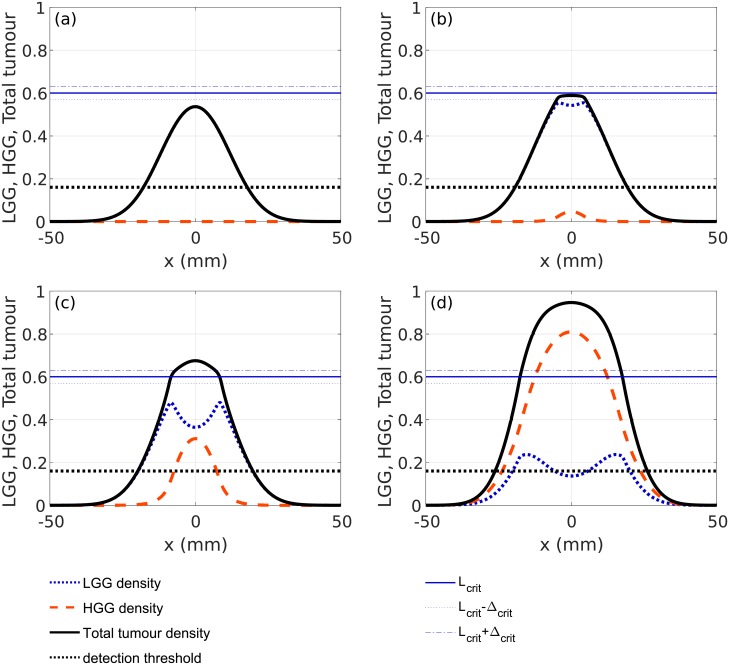
Snapshots of the evolution of the LGG and HGG cells densities solving system (1) for the parameter values and initial conditions as described in Sec. Evolution of virtual patients’ tumours. The densities of LGG cells *L*(*x*, *t*) (red dashed lines), HGG cells *H*(*x*, *t*) (blue dotted lines) and the total tumour (black solid line) are shown. The horizontal blue lines correspond to the value *L*_*crit*_ (solid line), *L*_*crit*_ − Δ_*crit*_ (blue dotted line) and *L*_*crit*_ + Δ_*crit*_ (blue dashed-dotted line). The value of the detection threshold is marked with dashed horizontal lines. Results are shown for time *t* equal (a) 12, (b) 20, (c) 22 and (d) 25 months.

The typical evolution of a virtual tumour governed by system (1) is presented in Figs 1 and 2. Parameters *h*_0_, *ρ*_*L*_, *D*_*L*_, *D*_*H*_ of the virtual patient were fixed to the mean values of parameters fitted to patients data, see Table 4. The initial condition for the simulation is

$$\begin{array}{c} H\left(x,0\right)=0,\;L\left(x,0\right)=h_{0}\exp(-x^{2}/235.012) \end{array}$$ (5)

with *x* measured in mm, what gives an initial tumour diameter of 31.278 mm, being the mean value of initial tumour diameters of patients selected for model fitting. The remaining parameters used in the simulations are fixed as listed in Table 1.

**Table 4:** Model parameters fitted for each patient and errors of fits.

patient id	*h*_0_	*ρ*_*L*_ (/day)	*D*_*L*_ (mm^2^/day)	*D*_*H*_ (mm^2^/day)	error
60	0.3404	0.001223	0.001227	0.004056	0.38%
61	0.3005	0.000253	0.000306	0.894292	0.21%
65	0.5435	0.000447	0.000858	0.745564	0.14%
66	0.5371	0.000243	0.000550	0.008817	0.08%
141	0.4613	0.001789	0.003597	0.015512	0.19%
165	0.5692	0.000553	0.0007558	0.001919	0.83%
195	0.4602	0.000764	0.007971	0.173277	0.45%
211	0.4144	0.002387	0.007383	0.087882	0.02%
mean (virtual patient)	0.4533	0.0009	0.0028	0.2414	0.2875%
st. deviation	0.0973	0.0008	0.0031	0.3639	0.2622%

When the cell density of LGG cells (Figs 1(a) and 2(a)) reaches the critical level (Fig 2(b)) HGG cells appear and start growing (Fig 2(c)) until they completely dominate the dynamics (Figs 1(b) and 2(d)). This change in a cellular density is observed in patients as an appearance or a significant increment in contrast-enhancing areas in post-contrast T1+Gd MRI scans in the areas where the malignant transformation occurs. It also causes a considerable increase in the total tumour mass that is reflected in solutions of our model, see Fig 2 and also visible in diffusion-weighted imaging in the form of a restriction of the water mobility in the corresponding tumour areas [56]. Moreover, after some time the tumour is almost completely composed of the high-grade tumour cells as observed in clinical practice and also reflected by our model.

### System (1) describes the dynamics of real-patients LGG growth and its malignant transformation

We fitted the solutions of the system (1) to the MRI longitudinal volumetric data for each patient included in the study as described in the methods section. Parameters values obtained are listed in Table 4.

**Fig 3:**
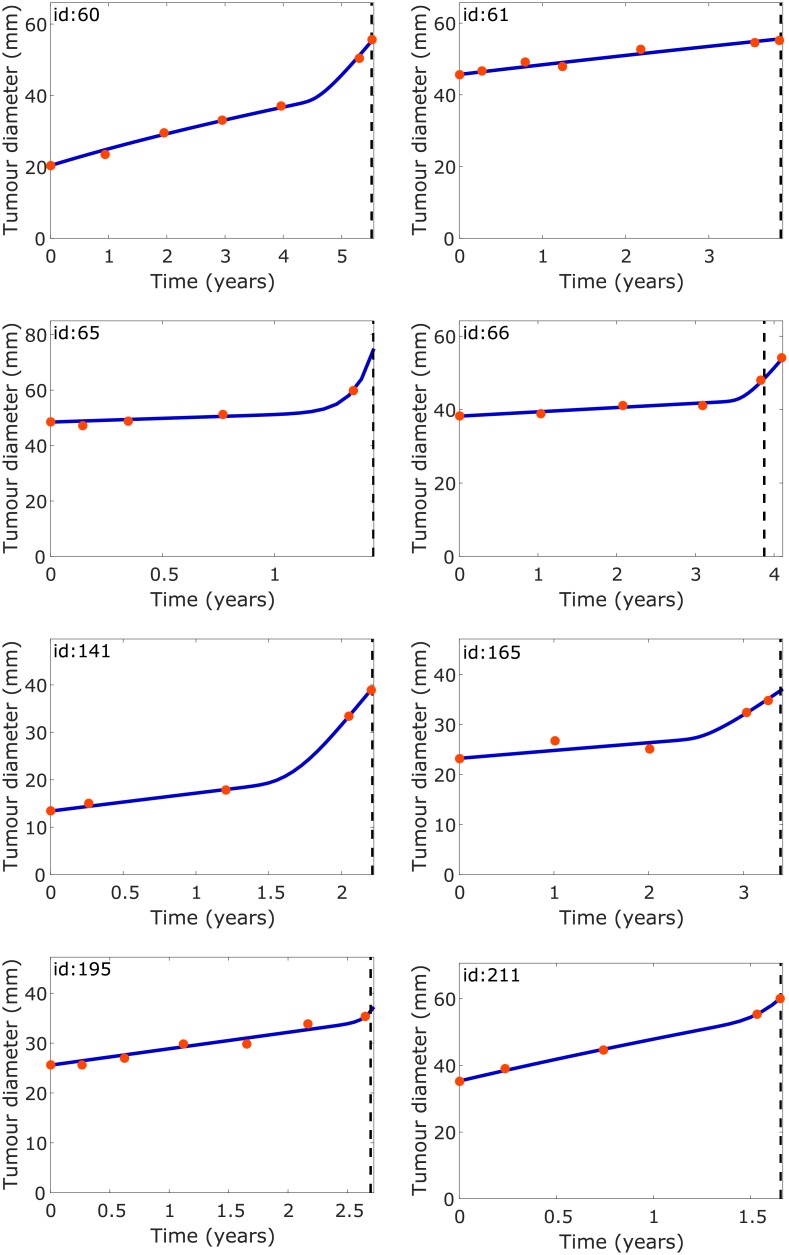
Tumour diameter evolution for patients with confirmed malignant transformation. The diameters calculated from MRI scans (red circles) and from the fitted mathematical model (1) (solid blue lines) are shown. The vertical black dashed lines mark the times when malignant transformation was confirmed histopathologically. The values of parameters *σ*, *h*_0_, *ρ*_*L*_, *D*_*L*_, *D*_*H*_ were different for each patient (see Table 4). The parameter *σ* was calculated using Eq (4). Other parameters values are listed in Table 1.

Fig 3 shows for each patient included in the study the real tumour diameter longitudinal data obtained from the MRI scans together with the virtual tumour evolution obtained with the parameters listed in Table 4. The model dynamics shows a very good agreement with the real dynamics despite the use of a minimal number of parameters.

### LGG proliferation rate determines prognosis

To correlate the numerical simulations with the patient prognosis we assume that a tumour of a certain size is not compatible with life as stated, among others, in [46, 48]. This critical size is usually referred to as fatal tumour burden. In this study we fix the value of the fatal tumour burden to be equal to the tumour of 8 cm in diameter. Although this is critically dependent on tumour location, in general this is believed to be a reasonable approximation. The assumed value of fatal tumour burden is larger than the value suggested in previous studies of HGG growth with the use of mathematical models [46, 48] due to the fact that in our patients database the tumours of diameter even greater than 7.5 cm are reported. Moreover, the slow evolution of LGGs allows the brain to remap neurological functions to other brain areas enabling these tumours to grow to larger sizes in comparison to HGGs. In our mathematical framework we refer to the time ranging from detection to the time when the virtual tumour reaches the fatal tumour burden size as virtual patient survival.

**Fig 4:**
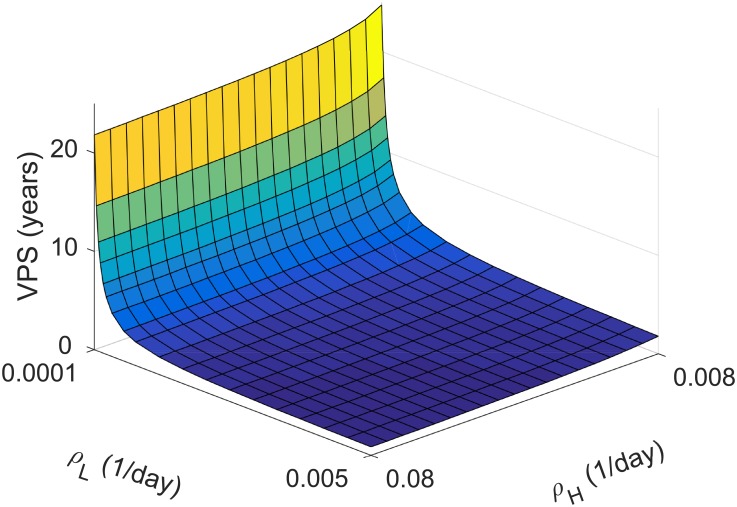
Virtual patient survival (VPS) for different proliferation rates of LGG and HGG cells evolving as indicated by system (1). The initial tumour cell densities and parameters for virtual patients were taken as in Sec. Evolution of virtual patients’ tumours.

Based on numerical simulations of system (1) we conclude that the parameter *ρ*_*L*_ has a large influence on virtual patient survival. Fig 4 presents virtual patient survival as a function of both proliferation rates for LGG and HGG in the absence of any treatment and parameters fixed to the mean values obtained from fit to real patients data. We observe that changes in *ρ*_*H*_ have a minor effect on survival. However, a modification of *ρ*_*L*_, proliferation rate in the slowly growing stage of the disease, affects very significantly the virtual patient survival.

For the mean value of proliferation rate *ρ*_*L*_ = 0.0009/day virtual patient survival varies from 3.7222 years (for *ρ*_*H*_ = 0.008/day) to 2.1944 years (for *ρ*_*H*_ = 0.08/day). For the typical value of *ρ*_*H*_ = 0.042/day (see Table 1) virtual patient survival varies from 22.2778 years (for *ρ*_*L*_ = 0.001/day) to 1.0556 year (for *ρ*_*L*_ = 0.008/day). This is an expected outcome of the model since in previous works [34, 52, 57, 58] it has been shown that the value of velocity of LGG growth is a prognostic factor for malignant transformation-free survival and overall survival. It is also reflected by our model.

This is an interesting result which can have an influence on treatment planing since in many cases more intensive therapies such as radiotherapy or even significantly less toxic chemotherapy are reserved until there are signs of progression (*e.g.* spots of contrast enhancement on T1+Gd MRI scans). Although an inclusion of treatment into the model and further analysis are needed, our results indicate that it is better to use more aggressive intervention earlier, trying to prevent malignant transformation than to wait and treat already transformed and fast-growing tumour cells. One can base the treatment decisions on the estimates of the tumour aggressiveness and potential time to malignant transformation which can be derived from imaging [29], taking into account also the levels of cytotoxicity induced. This is also in line with recent results that one may get a substantial therapeutical benefit by the use of protracted therapies instead of waiting for the malignant transformation to occur [30, 31].

### The rate of phenotypic change does not change survival significantly

**Fig 5:**
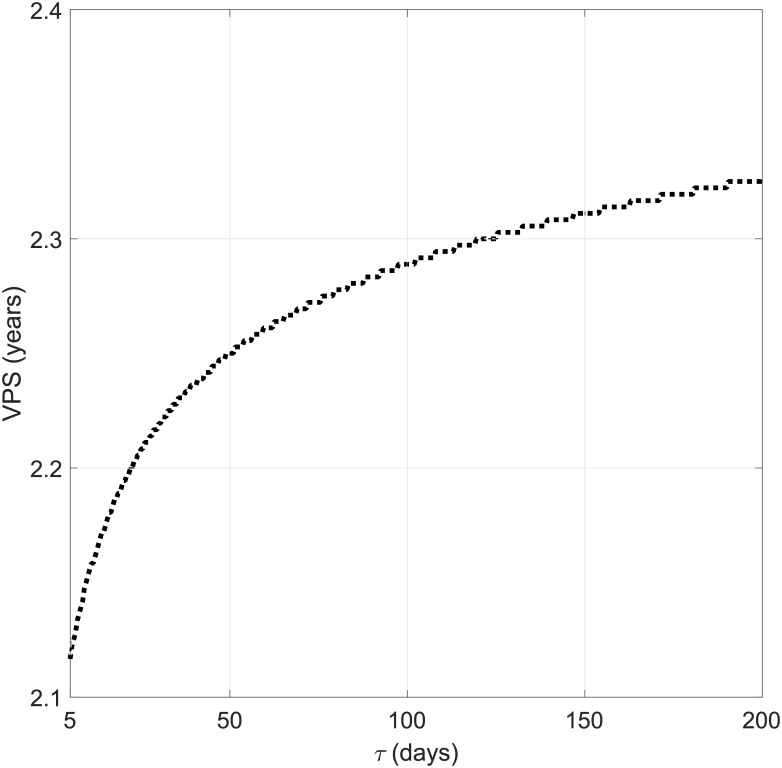
Relation between the characteristic time of phenotypic change *τ* and virtual patient survival. The initial tumour cell densities and parameters’ values are taken as in Fig 1.

Intuitively, time *τ* gives an order of magnitude of the time to complete malignant transformation once the density reaches the critical level. Fig 5 shows the dependence of the virtual patient survival on the parameter *τ* for our standard set of parameters described in Sec. Evolution of virtual patients’ tumours, which are the mean values obtained from fit to real patients data. It is clear that the choice of this parameter does not essentially influence survival which differs within the range of 3 months, which is not significant when compared to the average survival of low grade gliomas [13]. Since the major component of survival time is given by the survival before the malignant transformation, this time adds only weeks or at most a few months to the total survival. For the other sets of parameters the results were very similar.

### Theoretical estimates of LGG growth and malignant transformation

#### Estimates of LGG growth

Initially, until the onset of malignant transformation the tumour is composed only of LGG cells and thus its evolution is described by a single FKE:

$$\begin{array}{c} \frac{\partial L}{\partial t}=\rho_{L}L(1-\frac{L}{K})+D_{L}\Delta L \end{array}$$ (6a)

with initial condition:

$$\begin{array}{c} L\left(0,x\right)=h_{0}\exp(-\frac{x^{2}}{\sigma }) \end{array}$$ (6b)

and no-flux boundary condition:

$$\begin{array}{c} \frac{\partial L}{\partial n}{|}_{\partial \Omega }=0, \end{array}$$ (6c)

see system (1) and Eq (3). For convenience throughout this section we will use *ρ* and *D* instead of *ρ*_*L*_ and *D*_*L*_.

Until malignant transformation, glioma total density is significantly smaller than the maximal cellular density in brain. As a result, tumour cells do not have to compete for space. Therefore, we neglect in system (6) the non-linear term and approximate the solution *L* to system (6) by a solution *u* to the following equation referred to as Skellam equation [59]

$$\begin{array}{c} u_{t}=\rho u+D\Delta u \end{array}$$ (7)

together with free boundary condition and the initial condition

$$\begin{array}{c} u\left(0,x\right)=h_{0}\exp(-\frac{x^{2}}{\sigma }). \end{array}$$ (8)

Therefore, we approximate the tumour cell density after diagnosis by a solution of Eq (7):

$$\begin{array}{c} u\left(t,x\right)=h_{0}e^{\rho t}\sqrt{\frac{\sigma }{\sigma +4Dt}}e^{-\frac{x^{2}}{\sigma +4Dt}}. \end{array}$$ (9)

The virtual tumour is detectable at time *t* if max_*x*_ *u*(*t*, *x*) ≥ *d*. Then it is detectable for all positive times if

$$\begin{array}{c} \min_{t\geq 0}\max_{x}u\left(t,x\right)=\min_{t\geq 0}u\left(t,0\right)=\frac{\partial }{\partial t}u\left(t,0\right)\geq d. \end{array}$$

Function *u*(*t*, 0) is increasing if

$$\begin{array}{c} \sigma \rho >2D \end{array}$$ (10)

and minimum is attained for time $\frac{2D-\sigma \rho }{4D\rho }$. Finally when the condition

$$\begin{array}{c} h_{0}e^{\frac{2D-\sigma \rho }{4D}}\sqrt{\frac{\sigma \rho }{2D}}\geq d \end{array}$$

holds the analytical formula for the radius of tumour reads

$$\begin{array}{c} \begin{array}{cc} r(t)= & 2t\sqrt{D\rho }(1-\frac{\ln(\sigma +4Dt)}{2\rho t}+\frac{1}{t}(\frac{\sigma }{4D}+\frac{1}{\rho }\ln(\frac{h_{0}}{d}\sqrt{\sigma }))+\\ & \;\;\;-\frac{\sigma \ln(\sigma +4Dt)}{8D\rho t^{2}}+\frac{\sigma }{4D\rho t^{2}}\ln(\frac{h_{0}}{d}\sqrt{\sigma }){)}^{1/2}. \end{array} \end{array}$$ (11)

Next, calculating the first derivative of *r* with respect to time we obtain the tumour growth velocity:

$$\begin{array}{c} r^{\prime }\left(t\right)=\frac{2\sqrt{D\rho }(1-\frac{\ln(\sigma +4Dt)}{4\rho t}+\frac{1}{2t}(\frac{\sigma }{4D}+\frac{1}{\rho }\ln(\frac{h_{0}}{d}\sqrt{\sigma })-\frac{1}{2\rho }))}{\sqrt{1-\frac{\ln(\sigma +4Dt)}{2\rho t}+\frac{1}{t}(\frac{\sigma }{4D}+\frac{1}{\rho }\ln(\frac{h_{0}}{d}\sqrt{\sigma }))-\frac{\sigma \ln(\sigma +4Dt)}{8D\rho t^{2}}+\frac{\sigma \ln(\frac{h_{0}}{d}\sqrt{\sigma })}{4D\rho t^{2}}}}. \end{array}$$ (12)

Clearly, the formulae for the tumour radius and tumour growth velocity are rather complex. Thus, we derive approximations of these formulae for the case when *t* ≪ 1 and when *t* ≫ 1. First we investigate the long time behaviour of Eqs (11) and (12). In this case using Taylor expansion we have

$$\begin{array}{c} \ln\left(\sigma +4Dt\right)=\ln t+\ln\left(4D\right)+\ln(1+\frac{\sigma }{4Dt})=\ln t+\ln\left(4D\right)+\frac{\sigma }{4Dt}+o(\frac{1}{t}). \end{array}$$ (13)

Plugging Eqs (13) into (11), using asymptotic approximation and keeping only terms of order equal or higher than 1/*t* we obtain

$$\begin{array}{c} r\left(t\right)=2t\sqrt{D\rho }(1-\frac{\ln t}{4\rho t}+\frac{1}{2\rho t}(\frac{\sigma \rho }{4D}+\ln(\frac{h_{0}}{d}\sqrt{\sigma })-\frac{\ln4D}{2})+o(\frac{1}{t})). \end{array}$$ (14)

A similar procedure leads to the formula for the velocity:

$$\begin{array}{c} r^{\prime }\left(t\right)=2\sqrt{D\rho }(1-\frac{1}{4\rho }\cdot \frac{1}{t}+o(\frac{1}{t})). \end{array}$$ (15)

This results show that tumour radius asymptotically grows with the speed slower than the asymptotic velocity of FKE, where the first correction term is equal to 1/(4*ρt*). This means that for large times lack of restriction on the density leads to slower tumour growth. This might seem a surprising result, but in fact it not so much. Although density limitation due to the competition slower cell division it also forces cells to move to the area with less density leading to faster increase in tumour radius.

The behaviour of the radius and velocity of tumour for small times is even more important. The maximal tumour density is relatively small and we expect better agreement of the results with the full model. In order to obtain asymptotic approximation of the tumour radius for *t* ≪ 1 we expand the right-hand side of Eq (11) in Taylor series around *t* = 0. We have

$$\begin{array}{c} \ln\left(\sigma +4Dt\right)=\ln\sigma +\ln(1+\frac{4D}{\sigma }t)=\ln\sigma +\frac{4D}{\sigma }t+o\left(t\right),\;\text{as}\;t\to 0. \end{array}$$

Thus, neglecting the terms of order higher than *t* and using Eq (4) to eliminate ln(*h*_0_/*d*) we arrive at

$$\begin{array}{c} r\left(t\right)=r_{0}(1+\frac{1}{2}(\frac{4D}{\sigma }+\frac{\sigma \rho -2D}{r_{0}^{2}})t+o\left(t\right)). \end{array}$$ (16)

Finally using the same techniques we derive the approximation of the velocity of the tumour growth as *t* → 0:

$$r^{\prime }(t)=\frac{2D}{\sigma }r_{0}+\frac{\sigma \rho -2D}{2r_{0}}+t\left(\frac{4D}{\sigma }\cdot \frac{\sigma \rho -D}{r_{0}}-\frac{r_{0}}{4}{\left(\frac{4D}{\sigma }+\frac{\sigma \rho -2D}{r_{0}^{2}}\right)}^{2}\right)+o(t).$$ (17)

It is interesting that the velocity of the tumour growth depends on *r*_0_ or, being precise, on the term ln(*h*_0_/*d*). In particular for *σρ* > *D* there exists a ratio *h*_0_/*d* for which this velocity is minimal.

**Fig 6:**
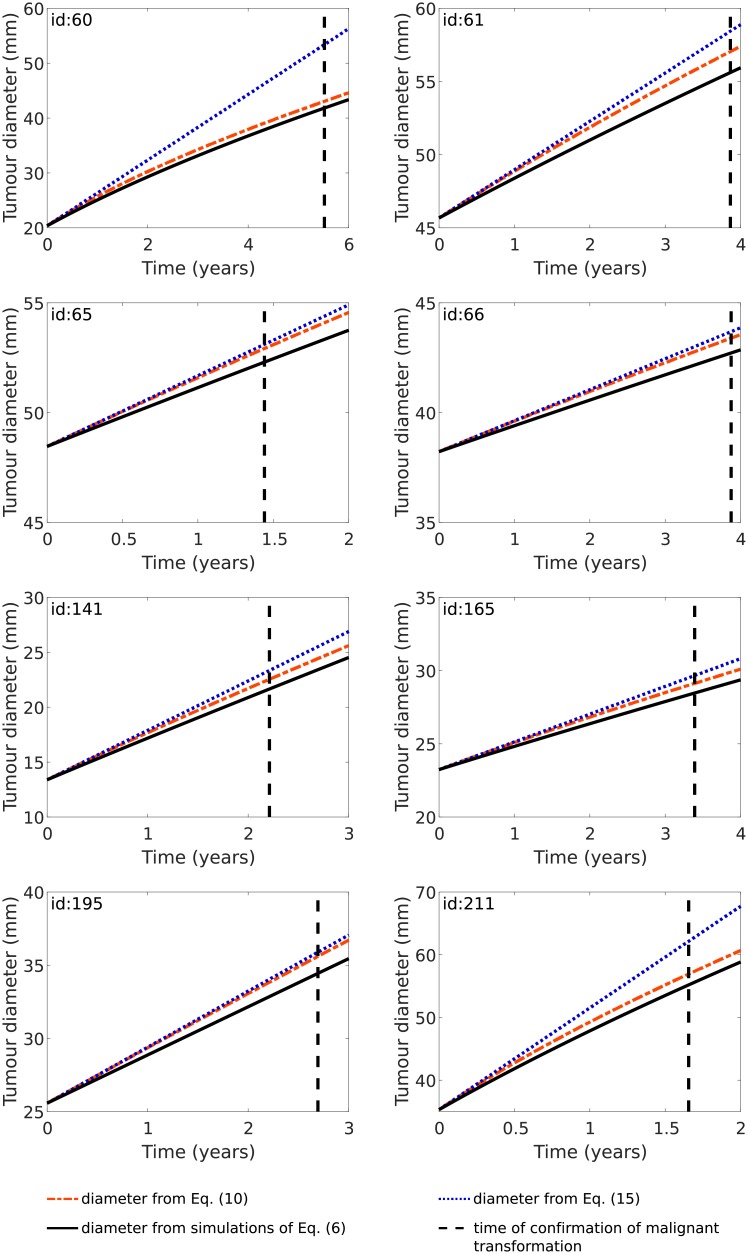
Evolution of LGGs diameter—Results based on the simulations of FKE Eq (6) (black solid line), analytic equation of radius evolution Eq (11) (red dashed-dotted line) due to Skellam model (7) and asymptotic behaviour of radius as *t* → 0 Eq (16) (blue dotted line). The vertical dashed line denotes the time when malignant transformation was confirmed histopathologically. The model parameters and initial conditions were the same as in Fig 3 for patients selected in this study.

It is easily seen that for *t* → 0 the value of tumour radius and tumour growth velocity tend to *r*_0_ and $\frac{2D}{\sigma }r_{0}+\frac{\sigma \rho -2D}{2r_{0}},$ respectively. We present the results of comparison of FKE, Skellam model and asymptotic behaviour in Fig 6 using the parameters values estimated for patients selected in this study.

#### Estimates of malignant transformation

Here we intend to provide some analytical estimates for the onset of malignant transformation as we believe that knowing the approximated time of this transformation could help in making clinical decisions [60].

The onset of malignant transformation can be estimated from numerical simulations of Eq (6), let us denote it as *t*_*OMT*_. However instead of considering partial differential equations, we would like to obtain algebraic formula feasible to solve in common programmes like Microsoft Excel, available in clinics.

Based on Eq (9) we can estimate the time *t*_*OMT*, *S*_ of the onset of malignant transformation as the time when the LGG cell density hits the value *L*_*crit*_ − Δ_*crit*_. Let us recall that *h*_0_ < *L*_*crit*_ − Δ_*crit*_, see Sec. Typical values and ranges of the model parameters. As function *u* attains its maximum in *x* = 0, we will calculate *t*_*OMT*_ in the following way:

$$\begin{array}{lll} L_{crit}-\Delta_{crit} & = & h_{0}{\text{e}}^{\rho t_{OMT,S}}\sqrt{\frac{\sigma }{\sigma +4Dt_{OMT,S}}},\\ 2\rho t_{OMT,S} & = & \text{ln}\left({\left(\frac{L_{crit}-\Delta_{crit}}{h_{0}}\right)}^{2}\left(1+\frac{4D}{\sigma }t_{OMT,S}\right)\right). \end{array}$$ (18)

The right-hand side of Eq (18) is a convex function of *t*_*OMT*_. The solution of Eq (18) exists when the condition

$$\begin{array}{c} \sigma \rho \geq 2D \end{array}$$

holds, compare condition (10).

We observe that *t*_*OMT*_ strongly depends on tumour density at center, that is where the cellular density is highest. For sufficiently small LGG cell diffusion coefficient we can approximate the evolution of tumour density at *x* = 0 by the logistic equation instead of solving nonlinear partial differential equation. Thus, we consider *L*(0, *t*) ≤ *u*(*t*), where *u* is a solution to

$$\begin{array}{c} u_{t}=\rho u(1-\frac{u}{K}) \end{array}$$ (19a)

with initial condition given by the density in the center of the tumour:

$$\begin{array}{c} u\left(0\right)=h_{0}. \end{array}$$ (19b)

Solving Eq (19), we obtain that onset of malignant transformation could be approximated as:

$$\begin{array}{c} t_{OMT,L}=\frac{1}{\rho }\ln(\frac{(L_{crit}-\Delta_{crit})\left(1-h_{0}\right)}{h_{0}(1-(L_{crit}-\Delta_{crit}))}). \end{array}$$ (20)

**Fig 7:**
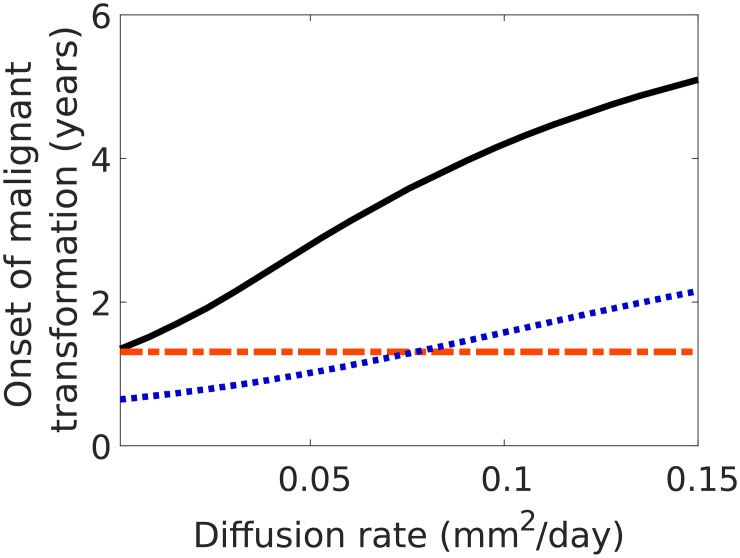
Estimates of the onsets of malignant transformation:*t*_*OMT*_ (black solid line), *t*_*OMT*,*S*_ (blue dotted line) and *t*_*OMT*,*L*_ (red dashed-dotted line) for different values of diffusion rate *D*. The initial tumour cell densities and other parameters’ values are taken as in Fig 1.

Clearly, such estimation is good for small diffusion rates of LGG cells, but on the contrary (for larger diffusion coefficients) estimation given by Eq (18) is a better one, compare Fig 7. Thus, we would propose to estimate *t*_*OMT*_ analytically as

$$\begin{array}{c} t_{OMT}\approx \max\{t_{OMT,S},t_{OMT,L}\}. \end{array}$$ (21)

**Fig 8:**
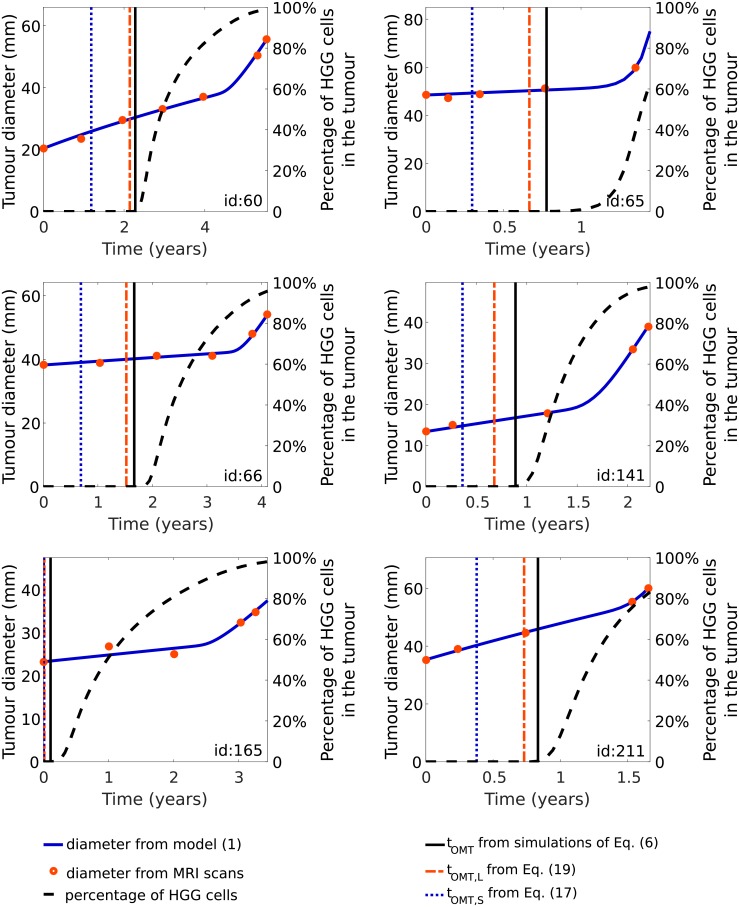
Evolution of tumour diameter due to system (1) together with the clinical data as in Fig 3, the estimation of the onset of malignant transformation calculated using simulations of Eq (6) (vertical black solid lines), Eq (20) (red dashed-dotted lines) and Eq (18) (blue dotted lines). We also show the percentages of HGG cells in total tumour mass calculated based on the results of simulations. The time scale of simulation ends when malignant transformation was confirmed histopathologically. Model parameters and initial conditions were the same as in Fig 3 for the first six patients in this study (dashed lines).

We have computed estimates of the onset of malignant transformation for first six patients for which the occurrence of malignant transformation was observable radiologically in tumour size, see Fig 8. Our work shows that all estimated onsets of malignant transformation appears in medically viable time period, see also S1 File. We can observe a significant delay from the onset of malignant transformation to the visible change in the velocity of tumour radius growth. A natural explanation for this is that there is a visible increase in detectable tumour size when the significant part of the tumour is formed by already transformed cells.

## Discussion

In this paper we addressed the process of malignant transformation of low-grade gliomas, what is the main reason for the disease lethality. The early detection of malignant transformation could improve the therapeutical management and prevent the misdiagnosis of actual tumour grade. It can have major therapeutic implications, namely the tumour with undetected malignant transformation would be treated less aggressively than necessary and as a result an increase in mortality will occur.

To our knowledge up to now the only mathematical model accounting for the LGG malignant transformation was formulated by Swanson *et al.* in [26]. In that work a system of differential equations was describing the evolution of three types of glioma cells (normoxic, hypoxic and necrotic) with a vascular component and antiangiogenic factors. Interestingly, authors claim that the accumulations of genetic mutations is not necessary for malignant progression and the growth kinetics parameters alone can drive the glioma transformation. The main drawbacks of that model are their complexity and the fact that some of the underlying biological assumptions that are not completely realistic. For instance, normoxic cells convert directly not only to hypoxic, but also to necrotic ones, which is contradictory to biological observations.

Recently it has been hypothesised that malignant transformation may be triggered by the change of the tumour microenvironment due to the elevation of the cell density in a specific tumour areas [29, 30, 33, 39]. In fact, previous studies suggested the use of antithrombotics to avoid early tumour-induced vaso-occlusions (probably caused by the increment in cellularity) in order to delay the malignant transformation [38].

In this paper for the first time we try to use this concept in a quantitative way to describe the full process of the malignant transformation from a LGG to a HGG. We describe this process in a minimal way using a model of two coupled FKEs in which total tumour density is a driving force of phenotypic change. Interestingly, the model is able to reproduce the main features of the transition from low grade into high grade glioma. We presume that in our mathematical framework we can treat the tumour consisting of both LGG and HGG cells (see Fig 6) as WHO grade III glioma, which is an intermediate tumour stage between LGG (grade II) and secondary glioblastoma (grade IV) both histologically and in molecular features [61, 62]. Grade III gliomas, compared to grade II tumours, are more cellular, demonstrate more atypia, and mitoses are seen. However unlike glioblastomas, they lack vascular proliferation and necrosis on pathologic evaluation. The difference between gliomas grade III and IV is also reflected in the patients overall survival [60, 63] and prognosis, implying the necessity of using different treatment strategy.

We were also able to fit the model to the retrospective volumetric data of LGG patients who underwent malignant transformation and obtained a very good agreement. Based on this results we can treat our new model as a first step to the investigation of malignant progression as a function of patient-specific coefficients. We suggest combining the use of the early imaging and the results of mathematical modelling. To be specific, first we simplified our model of evolution of LGGs and obtained analytical equations for tumour radius and velocity before the onset of malignant transformation. As a result, we were able to provide an early approximation of the onset of malignant transformation based on the patient-specific parameters. Although Eq (21) looks complex, this formula is a significant simplification as instead of considering in fact a system of partial differential equations, we deal with an algebraic equation. Based on this formula and retrospective volumetric patients data, we have been able to compute *post-hoc* estimates of the onset of malignant transformation using the values of fitted parameters for individual patients. All estimated onsets of malignant transformation appears in medically viable time period, however there have been a significant delay from these times to the visible change in the velocity of tumour radius growth suggesting that there is a visible increase in detectable tumour size when the already transformed cells form the substantial part of the tumour. Importantly, the obtained values do not overestimate medically confirmed malignant transformation time. Thus, we can interpret the estimated values as the earliest times when malignant transformation could occur.

Let us note that the estimated time of onset of malignant transformation depends crucially on three biologically relevant parameters: proliferation rate, motility rate and mean initial density. The last one is essential to estimate in an unambiguous way the time of malignant transformation before it occurs. In general, we would like to provide predictions of time to malignant transformation for individual patients using data of only few medical examinations (MRI scans). However, our research shows that in order to do so, we would need not only volumetric data, but also imaging data at least from the first diagnostic MRI, based on which an initial tumour density could be estimated. Clearly, the proliferation and diffusion rates for LGG cells could be estimated from a few MRIs (possibly three) using *e.g.* a standard linear regression method. Subsequently, having parameters describing initial LGG density and tumour growth rates, the estimate of malignant transformation time can be computed from Eq (21) using standard Microsoft Excel package, which, in contrast to considering full partial differential model, could be done even in clinics. In such a way, combining the modelling approach and imaging, one would be possibly able to predict non-invasively the malignant transformation. Interestingly, Hathout *et al.* in [52] using the methodology from the previous works, *e.g.* [36], estimated mean proliferation and motility rate based on results of two MRIs for contrast-enhancing grade II astrocytoma and found out that those kinetics rates were significantly higher in the tumours that transformed to grade III or IV gliomas. It has been hypothesised recently that using different MRI modalities one is able to identify patients which tumours has recurred and underwent malignant transformation [64] as well as predict if the risk of rapid malignant transformation is large [22, 23, 65]. The use of perfusion-weighted MRI should be considered for broader use due to its potential [22, 23, 65]. We believe that by continuing the research on both advances in analysis of imaging modalities and mathematical modelling we would also be able to predict successfully malignant transformation.

Using such approach, the results obtained from imaging data and fitting the model opens the doors for the treatment personalisation. The estimations of the time of malignant transformation can possibly assist in selecting the best treatment. In particular, cases of fast-growing tumours or those which initial density was found to be significant should be followed with imaging thoroughly and early treatments strategies should be taken into account. One could consider applying treatment in the time of predicted malignant transformation. By eradicating glioma cells either by surgery or chemotherapy, the tumour cells density will be reduced resulting in prolonged malignant transformation-free survival and as a consequence overall survival, as well. In the future we would like to address the problem of optimal therapeutical schedulings taking into account the results of this study. In order to do so, the mathematical model may be improved by inclusion of more biological details.

We also believe that further understanding of the dynamics of the malignant transformation of LGGs may enable the development of more effective treatment strategies aimed at prolonging recurrence and delaying the arising of malignancy. Thus, further studies aimed at improving the understanding of the evolution of the malignant transformation, coupling multimodal imaging with mathematical models and studying the impact of optimised therapeutical schedules on the time to malignant transformation are necessary.

## Supporting information

S1 File Patients data. Containing data of LGG patients followed at Bern University Hospital selected in the study, see also Sec. Patients data.
(XLSX)
